# Neurosurgical Management of a Thoracic Dorsal Arachnoid Web: Case Illustration

**DOI:** 10.7759/cureus.4945

**Published:** 2019-06-19

**Authors:** Arvin R Wali, Harjus S Birk, Joel Martin, David R Santiago-Dieppa, Joseph Ciacci

**Affiliations:** 1 Neurosurgery, University of California, San Diego, La Jolla, USA

**Keywords:** arachnoid web, intradural extramedullary lesions, arachnoid band, scalpel sign

## Abstract

Dorsal thoracic arachnoid webs are rare clinical entities caused by a thickened intradural extramedullary band of arachnoid tissue that compresses the spinal cord, and often present with progressive back pain, paresthesias, and lower extremity weakness. In this report, we review the radiographic features of the “Scalpel Sign” and describe the case of a 47-year-old male that failed conservative therapy and was found to have dorsal thoracic arachnoid web. The patient underwent laminectomy and microsurgical release of the compressing arachnoid band. Postoperatively, the patient had complete resolution of his pain. Intraoperatively, the somatosensory evoked potentials were improved once the band was released. The prompt diagnosis of dorsal arachnoid webs remains critical because surgical treatment arrests and potentially reverses the pathology.

## Introduction

Spinal arachnoid webs are rare clinical entities [1, 2] that are distinct from arachnoid cysts. Structurally they are comprised of a band of thickened arachnoid tissue that exerts locoregional mass-effect on the spinal cord [1, 3]. Spinal arachnoid webs have a characteristic appearance on magnetic resonance imaging (MRI) and computed tomography (CT) myelography that has been termed the “Scalpel Sign.” Prior reports have found that arachnoid webs frequently present with progressive back pain and myelopathy. Moreover, these lesions are commonly located within the dorsal thoracic spinal cord. Spinal arachnoid webs may also copresent with syringomyelia [4]. Surgical treatment of spinal arachnoid webs includes careful microdissection to remove the arachnoid web and decompress the indented spinal cord. Within this case report, we describe the diagnosis and operative management of a dorsal arachnoid web in a 49-year-old male who presented with three years of progressive back pain, without radicular features or neurologic weakness. This surgical intervention also utilized intraoperative neuro-monitoring, providing one of the first descriptions of intraoperative changes in somatosensory evoked potentials (SSEP).

## Case presentation

**Figure 1:**
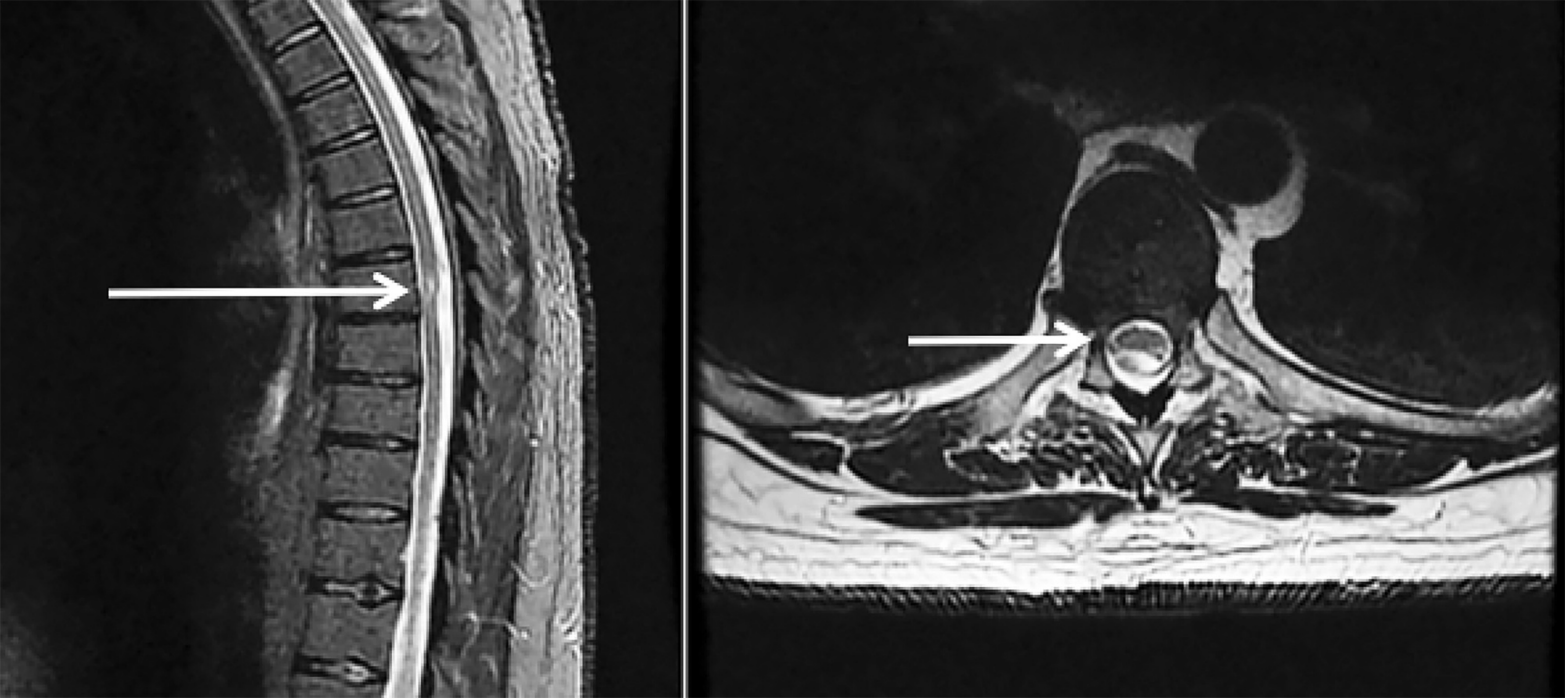
Sagittal and Axial T2 magnetic resonance imaging (MRI) demonstrating dorsal cord indentation at T7-8 concerning for possible "scalpel" sign.

A 47-year-old male presented to our institution with persistent, progressive low back pain, without any dermatomal radiculopathy, lower extremity weakness or paresthesias, and without point tenderness. The patient failed several weeks of conservative treatment with analgesics and physical therapy, and was then considered for further workup. He then underwent thoracic MRI and was found to have a positive “Scalpel Sign” on T2 weighted sequencing at the T7-T8 level suggesting an intradural extramedullary band of arachnoid tissue causing dorsal thoracic cord indentation, consistent with symptomatic dorsal arachnoid web (Figure 1).

Given the persistent, progressive nature of his back pain and an identified surgical target, the patient was offered surgery to excise the dorsal arachnoid web and relieve the locoregional mass effect that this band of tissue was exerting upon the spinal cord.

Operative report

**Figure 2:**
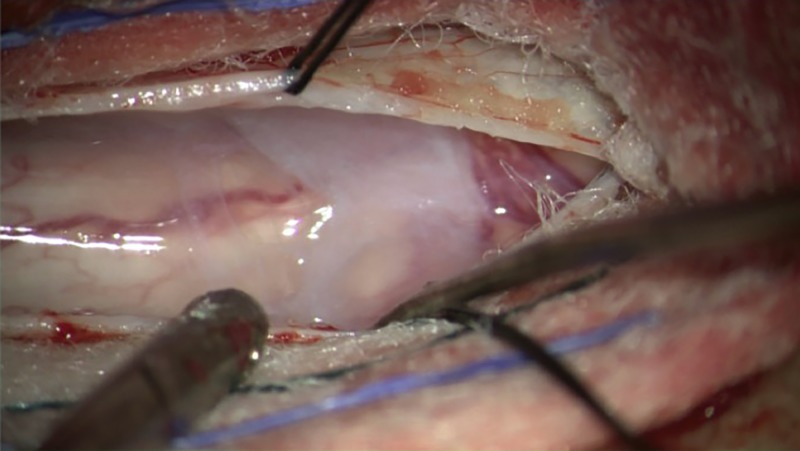
Intraoperative visualization of the arachnoid web upon durotomy of T8-T9 to expose the arachnoid web.

The patient was positioned prone, using a regular table. Neuromonitoring was available for the entirety of the case, and fluoroscopy was utilized to confirm localization of the T7-T8 level. The spinous processes and lamina of T7-T8 were exposed, and a laminectomy was performed. The dura was then opened sharply, exposing the dorsal spinal cord and revealing the arachnoid web (Figure 2).

The arachnoid web was then carefully microdisected away from the dorsal spinal cord and was removed in entirety. There was an immediate improvement in dorsal column SSEP after the dorsal web was lysed. The dura was then approximated with watertight closure and a post-surgical drain was placed.

Post-operative course

The patient remained at his neurologic baseline post-operatively, having full strength in his upper and lower extremities. There was no loss of sensation postoperatively; however, the patient had immediate relief from his low back pain. The drain was then removed, and the patient underwent a routine postoperative course with physical therapy and occupational therapy, prior to successful discharge to home. With the improvement in his low back pain, the patient no longer required the use of oral analgesia to rectify his pain as of clinic follow-up one month after surgery.

## Discussion

While rare, spinal arachnoid webs represent a unique anatomic anomaly that may cause pain, weakness, and progressive symptomology. Within the literature, surgical release of the compressing arachnoid band remains the gold standard for treatment and has the potential for cure. Consistent with the reported literature, our patient had a positive “Scalpel Sign” which assisted in the diagnosis and determination of an operative plan to resect the arachnoid web.

This report also describes the use of intraoperative neuromonitoring of SSEP during the laminectomy and excision of the arachnoid web. Within this operative case, the improvement in SSEP was consistent with the concurrent decompression of the indented spinal cord, serving as a proxy to assess the extent of decompression and may also serve as a predictor for a successful clinical outcome of decompressing the large diameter fibers of the posterior columns after surgery [5, 6].

Dorsal thoracic arachnoid web is a diagnosis that can be confirmed on MRI and/or CT myelography and treated with excellent clinical outcome with thorough microdissection. Further studies are required to understand the pathophysiology of the development of arachnoid webbing, why this lesion tends to be within the thoracic spine, and predictive techniques to identify which patients are more likely to develop syringomyelia in addition to the arachnoid webbing. Education on the “Scalpel Sign” should be incorporated within radiographic training of identifying arachnoid webbing as a radiologic feature that is consistent with arachnoid banding that is causing indentation within the thoracic spinal cord.

## Conclusions

This case further supports the use of the “Scalpel Sign” on MRI to diagnose dorsal arachnoid web and demonstrates surgery as the safe and definitive treatment for painful symptoms caused by indentation of the thoracic spinal cord. Decompression of the cord may cause immediate improvement in SSEP, providing immediate relief for patients with dorsal arachnoid web-induced back pain.
